# Unregistered Trials Are Unethical

**DOI:** 10.1371/journal.pmed.0020048

**Published:** 2005-02-22

**Authors:** Eswar Krishnan

**Affiliations:** Arthritis and Osteoporosis CenterReading, PennsylvaniaUnited States of America

Current journal requirements forcing clinical trials to be registered [1] are insufficient and are unlikely to solve the problem of negative trials never even making it to a journal. Most of the patients consenting to clinical trials do so out of altruism. It is a great betrayal of their trust to suppress clinical trial data. I suggest that institutional review boards refuse to allow human experimentation unless the protocol is filed in a central (online) repository. The primary data should also be required to be in the public domain (say, within 1–2 years after completion). Data obtained by appealing to altrusitic instincts, similar to money in public charities, are not proprietary information, nor can physicians cash out the trust of their patients. In reality, it is the pharmaceutical industry that stands to gain the most if data are made public as such data inform future research and help smaller, innovative companies avoid redundancy. Voluntarily sticking to higher standards of ethics will raise societal respect for the industry (currently being battered for greed) and attract a more talented workforce, and may even help the current efforts to reform the tort law.
